# Membrane Receptor-Induced Changes of the Protein Kinases A and C Activity May Play a Leading Role in Promoting Developmental Synapse Elimination at the Neuromuscular Junction

**DOI:** 10.3389/fnmol.2017.00255

**Published:** 2017-08-09

**Authors:** Josep M. Tomàs, Neus Garcia, Maria A. Lanuza, Laura Nadal, Marta Tomàs, Erica Hurtado, Anna Simó, Víctor Cilleros

**Affiliations:** Unitat d’Histologia i Neurobiologia (UHN), Facultat de Medicina i Ciències de la Salut, Universitat Rovira i Virgili Reus, Spain

**Keywords:** motor end-plate, postnatal synapse elimination, acetylcholine release, muscarinic acetylcholine receptors, adenosine receptors, neurotrophins, PKC, PKA

## Abstract

Synapses that are overproduced during histogenesis in the nervous system are eventually lost and connectivity is refined. Membrane receptor signaling leads to activity-dependent mutual influence and competition between axons directly or with the involvement of the postsynaptic cell and the associated glial cell/s. Presynaptic muscarinic acetylcholine (ACh) receptors (subtypes mAChR; M_1_, M_2_ and M_4_), adenosine receptors (AR; A_1_ and A_2A_) and the tropomyosin-related kinase B receptor (TrkB), among others, all cooperate in synapse elimination. Between these receptors there are several synergistic, antagonic and modulatory relations that clearly affect synapse elimination. Metabotropic receptors converge in a limited repertoire of intracellular effector kinases, particularly serine protein kinases A and C (PKA and PKC), to phosphorylate protein targets and bring about structural and functional changes leading to axon loss. In most cells A_1_, M_1_ and TrkB operate mainly by stimulating PKC whereas A_2A_, M_2_ and M_4_ inhibit PKA. We hypothesize that a membrane receptor-induced shifting in the protein kinases A and C activity (inhibition of PKA and/or stimulation of PKC) in some nerve endings may play an important role in promoting developmental synapse elimination at the neuromuscular junction (NMJ). This hypothesis is supported by: (i) the tonic effect (shown by using selective inhibitors) of several membrane receptors that accelerates axon loss between postnatal days P5–P9; (ii) the synergistic, antagonic and modulatory effects (shown by paired inhibition) of the receptors on axonal loss; (iii) the fact that the coupling of these receptors activates/inhibits the intracellular serine kinases; and (iv) the increase of the PKA activity, the reduction of the PKC activity or, in most cases, both situations simultaneously that presumably occurs in all the situations of singly and paired inhibition of the mAChR, AR and TrkB receptors. The use of transgenic animals and various combinations of selective and specific PKA and PKC inhibitors could help to elucidate the role of these kinases in synapse maturation.

## Developmental Axonal Loss and Synapse Elimination

**Figure 1:**
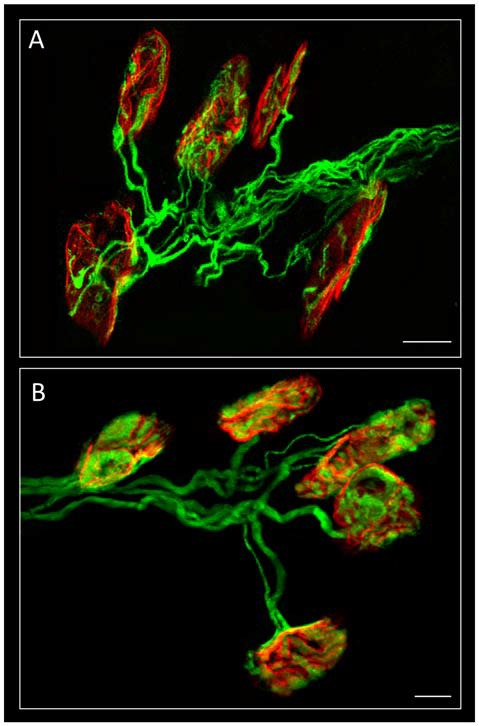
Confocal immunofluorescence. The image shows mono-innervated and polyinnervated synapses from C57BL/6J P7 **(A)** and YFP **(B)** control mice. The Levator auris longus muscle (LAL) neuromuscular junctions (NMJs) (in **A**) show the axons stained green by 200-kD neurofilament antibody and the postsynaptic nicotinic acetylcholine receptors (nAChR) clusters (both in **A,B**) stained red by TRITC-α-BTX. Scale bar: 10 μm. Image **(A)** has been adapted from Figure 1 in the original article (Tomàs et al., 2017). The original article is an open access article distributed under the terms of the Creative Commons Attribution License (http://creativecommons.org/licenses/by/2.0), which permits unrestricted use, distribution and reproduction in any medium, provided the original work is properly cited.

When the nervous system develops, the neurons and synapses involved in circuitry wiring and connectivity are overproduced. However, Hebbian competition between nerve processes and endings eliminates redundant synapses and refines the specificity of the functional circuits (Purves and Lichtman, 1980; Jansen and Fladby, 1990; Sanes and Lichtman, 1999). Synapses are lost throughout the nervous system during histogenesis (Bourgeois and Rakic, 1993). In the visual system, thalamocortical axons disconnect from cortical layer IV cells (Hubel et al., 1977; Huberman, 2007), in the cerebellum, climbing fibers disconnect from Purkinje cells (Daniel et al., 1992; Hashimoto and Kano, 2005) and in autonomic ganglia, preganglionic inputs disconnect from ganglion cells (Lichtman, 1997). Developmental axonal loss also occurs in neuromuscular junction (NMJ), the paradigmatic model of neuroscience. Most axonal elimination occurs during the first 2 weeks after birth. At birth, the NMJs are initially polyinnervated but, by the end of the axonal competition, the motor endplates are innervated by a solitary axon (Benoit and Changeux, 1975; O’Brien et al., 1978; Liu et al., 1994; Ribchester and Barry, 1994; Nguyen and Lichtman, 1996; Chang and Balice-Gordon, 1997; Sanes and Lichtman, 1999; Herrera and Zeng, 2003; Nelson et al., 2003; Wyatt and Balice-Gordon, 2003; Buffelli et al., 2004; Figure 1).

## Membrane Receptors in Axonal Loss

Membrane receptor signaling can play a role in axonal competition by allowing the various nerve endings to have an activity-dependent influence on one another directly or with the involvement of the postsynaptic cell and the associated glial cell/s (Keller-Peck et al., 2001; Tomàs et al., 2014). We observed that presynaptic muscarinic acetylcholine receptors (mAChR; subtypes M_1_, M_2_ and M_4_), adenosine receptors (AR; A_1_ and A_2A_) and the neurotrophin receptor (NTR) tropomyosin-related kinase B receptor (TrkB) all cooperate in the developmental synapse elimination process at this synapse [NMJ from the *Levator auris longus*—LAL—muscle of the B6.Cg-Tg (Thy1-YFP)16 Jrs/J mice (hereinafter YFP mice), and from C57BL/6J P7 mice] by favoring axonal competition and loss (Nadal et al., 2016a,b, 2017; Tomàs et al., 2017). Other receptors, for example glutamate receptors at the mice NMJ (Waerhaug and Ottersen, 1993) may collaborate because developmental synapse loss is slowed by reducing activation of the glutamate-NMDA receptor pathway (Personius et al., 2016).

**Figure 2:**
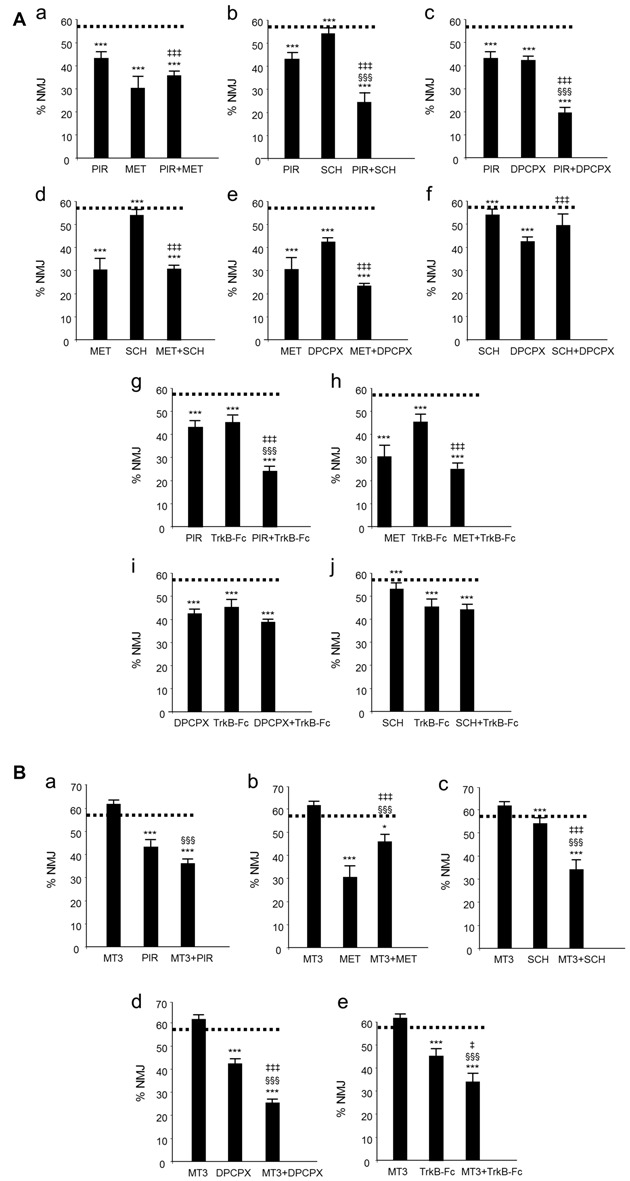
Changes in polyneuronal innervation of the NMJ after inhibiting the muscarinic ACh autoreceptors (mAChR), adenosine receptors (AR) and the tropomyosin-related kinase B receptor (TrkB) signaling in the YFP mice. **(Aa–j)** shows the percentage of monoinnervated NMJs in controls (PBS, dotted lines) and after exposure (four applications, one application every day after P5) to one inhibitor or after simultaneous inhibition of two receptors that individually affect axon loss (all inhibitors but MT3). The associations of MT3 with the other substances are represented in **(Ba–e)**. The symbols indicate: *P < 0.05, ***P < 0.005 when the corresponding antagonist or combinations of two substances are compared with control PBS. ^§§§^*P* < 0.005 when the combination of two substances is compared with the first substance. ^‡^*P* < 0.05, ^‡‡‡^*P* < 0.005 when the combination of two substances is compared with the second. The selective inhibitors are: methoctramine (MET), M_2_ inhibitor; pirenzepine (PIR), M_1_ inhibitor; 8-Cyclopentyl-1,3-IP3, inositol triphosphate (DPCPX), A_1_ inhibitor; SCH58261, A_2A_ inhibitor and inhibitor recombinant human TrkB-Fc Chimera (TrkB-Fc), TrkB inhibitor: this figure has been adapted and redrawn from Figures 3,4 in the original article by Nadal et al. (2016a). The original article is an open access article distributed under the terms of the Creative Commons Attribution License (http://creativecommons.org/licenses/by/2.0), which permits unrestricted use, distribution and reproduction in any medium, provided the original work is properly cited. The paired inhibition data of the AR and TrkB shown in the histograms i and j have not been previously published.

We have used the term cooperation above to define the collaboration between mAChR, AR and TrkB receptor pathways in controlling axonal loss. Cooperation requires the receptors to work together: (i) additively or synergistically; or (ii) occlusively or antagonically. We simultaneously applied two inhibitors (two selective antagonists from two different receptors) to reveal the possible additive or occlusive crosstalk effects between the corresponding pathways. The histograms in Figure 2 show the individual and the paired effects of these inhibitors on axonal loss at P9 (percentage of the monoinnervated synapses after exposure to blockers (data drawn from previous studies: Nadal et al., 2016a,b, 2017; Tomàs et al., 2017). The paired inhibition data of the AR and TrkB shown in histograms i and j from Figure 2A have not been previously published).

## Synergistic and Antagonic Effects of The mAChR, AR and TrkB That Affect Developmental Synapse Elimination

**Figure 3:**
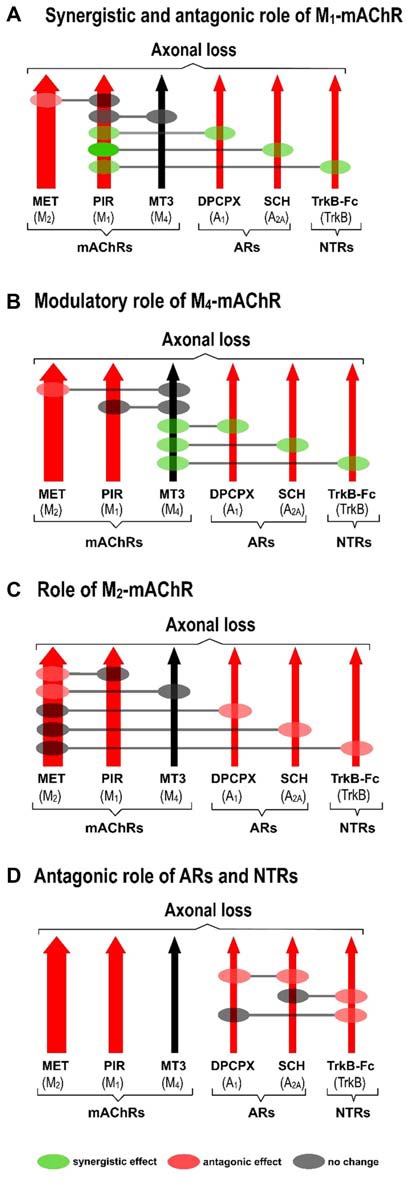
Cooperation between mAChR, AR and TrkB receptors. All diagrams **(A–D**; redrawn from previous work, except some unpublished data in diagram **(D)**, Nadal et al., 2016b, 2017), show the effect of the selective inhibitors in order of their ability to finally delay monoinnervation and keep a high percentage of synapses innervated by two or more axons (MET, M_2_ inhibitor; PIR, M_1_ inhibitor; DPCPX, A_1_ inhibitor; SCH58261, A_2A_ inhibitor; TrkB-Fc, TrkB inhibitor). The red arrows show how effective the blockers are at delaying elimination (the thicker they are, the greater their effect). Only the M_4_ blocker MT3 is unable to change axonal loss (black arrow in the figures). Diagrams show the cooperation links between the receptors as judging by the effect of the corresponding paired inhibition (gray circles mean there is no change, green circles mean there is a synergistic effect and red circles mean there is an antagonic effect between the receptors).

The receptors (Figure 2A) with the exception of the M_4_ subtype (Figure 2B), directly accelerate axon loss at P9 (when selectively blocked between P5 and P8, axonal elimination is reduced and this shows their tonic effect in normal conditions). All diagrams in Figure 3 (taken from previous articles, except some unpublished data in Figure 3D, see below; Nadal et al., 2016b, 2017), show the effect of the selective inhibitors in order of their ability to finally delay monoinnervation and keep a high percentage of synapses innervated by two or more axons (methoctramine (MET), M_2_ inhibitor; PIR, M_1_ inhibitor; 8-Cyclopentyl-1,3-IP3, inositol triphosphate (DPCPX), A_1_ inhibitor; SCH58261, A_2A_ inhibitor; inhibitor recombinant human TrkB-Fc Chimera (TrkB-Fc), TrkB inhibitor). The red arrows show approximately how effective the selective blockers are at delaying axonal elimination (the thicker they are, the greater their effect, although their absolute pharmacological potency cannot be directly compared). In this case, only the M_4_ blocker MT3 is unable to significantly change the percentage of monoinnervation (see the data in Figure 2B), which shows that there is no direct effect of M_4_ on axonal loss at this time (black arrow in Figures 3A–D).

Diagrams also show the cooperation links between the receptors as judging by the effect of the corresponding paired inhibitors exposition (gray circles mean there is no change, green circles mean there is a synergistic effect and red circles mean there is an antagonic effect).

### Synergistic Role of the M_1_ Subtype

Figure 3A shows the synergistic role of the M_1_ mAChR, which potentiates the effect of both AR (A_1_, 58% and A_2A_ 36%) and TrkB (25%) on axonal elimination. Only a small antagonic effect is observed on the potent M_2_ function and in this case the final effect is no different from the individual M_1_ effect on axon loss.

### Modulatory Role of the M_4_ Subtype

This receptor is not directly involved in axonal loss. Figure 3B shows, however, that it strongly potentiates the effect of AR (A_1_, 33% and A_2A_ 32%) and TrkB (23%) and also slightly inhibits the potent M_2_ effect. In fact, although M_4_ does not act directly by itself, its regulatory functions are similar to those of the M_1_ subtype. Therefore, M_4_ has a modulatory function. We think that though insufficient to promote an effect by itself, the M4 pathway may realize some priming action on the other pathways to facilitate them (the AR and the TrkB pathways) or to obstruct them (the M2 pathway).

### Role of M_2_ Subtype

M_2_ has a powerful effect on axon loss and only the other mAChRs, M_1_ and M_4,_ can slightly reduce its potency (Figure 3C).

### Antagonic Effects between AR and TrkB

Figure 3D shows that when the inhibitor recombinant human TrkB-Fc Chimera (TrkB-Fc) is associated with one of the AR inhibitors DPCPX or SCH58261, the effect is just the same as the individual effect of one of them on axon loss (in the graph, we have chosen to represent the position of the red circles only on the TrkB pathway for purposes of simplicity. These data have not been previously published). When both AR are blocked simultaneously, occlusion is complete and the final result is no different from that of the untreated control.

Thus, several synergistic, antagonic and modulatory relations are clearly observed between the receptors, which affect synapse elimination.

## Serine Kinases in Axonal Loss

Metabotropic membrane receptors converge in a limited repertoire of intracellular effector kinases (mainly serine protein kinases A and C [PKA and PKC]) to phosphorylate protein targets and bring about structural and functional changes that lead to axon loss. The nerve endings that lose the competitive process progressively weaken by diminishing the quantal content of the evoked ACh release in parallel with the progressive loss of nicotinic acetylcholine receptors (nAChR) from the postsynaptic muscle cell (Caulfield, 1993; Felder, 1995; Caulfield and Birdsall, 1998; Nathanson, 2000; Lanuza et al., 2001, 2002; Santafé et al., 2004; Garcia et al., 2010; Tomàs et al., 2014). Receptors and kinases may regulate coordinately these changes.

In the postsynaptic component, the phosphorylation of the nAChR delta and epsilon (delta nicotinic acetylcholine receptor subunit (nAChRδ) and epsilon nicotinic acetylcholine receptor subunit (nAChRε)) subunits may help the nAChR cluster to mature, which may also affect synapse loss during postnatal development. nPKCθ produces nAChR instability and loss by phosphorylating the delta subunit, while PKA reverses this effect and increases receptor stability by phosphorylating the epsilon subunit. Moreover, PKA and PKC may phosphorylate differently the nAChR in the different axon terminals (with different activity) that are in competition in the same synaptic site. PKC-induced dispersion under the weakest nerve terminals and a PKA-induced catching and stabilization under the more active axon terminals results in the differentiation of the postsynaptic gutters (Nelson et al., 2003; Lanuza et al., 2006, 2010, 2014). Also, protein phosphorylation is an important posttranslational modification of group I metabotropic glutamate receptors. Evidences indicate that PKA and PKC directly interact with mGluR1/5, phosphorylate specific serine or threonine sites and thereby regulate trafficking, distribution, and function of phosphorylated receptors (Mao and Wang, 2016).

In the presynaptic component, intracellular serine kinases, both PKA and PKC in the nerve terminals, could be directly involved in modulating calcium-dependent ACh release at the NMJ (Santafé et al., 2006, 2007a,b, 2009b; Tomàs et al., 2011). Specifically, PKC [alpha protein kinase C isoform (cPKCα), beta I protein kinase C isoform (cPKCβI) and epsilon protein kinase C isoform (nPKCε) isoforms are the candidates (Besalduch et al., 2010; Lanuza et al., 2010; Obis et al., 2015)] is able to reduce the ACh release capacity of the weak axons in developing polyinnervated synapses (Santafé et al., 2003, 2004, 2007b, 2009a,b; Tomàs et al., 2011). This effect on transmitter release may also be related with axonal loss because the competitive force of these nerve endings decreases.

In other molecular mechanisms PKA and PKC can phosphorylate the same molecule in different residues. For instance, SNAP25 is phosphorylated by PKA (in T138) and PKC (in S187) whereas Munc18 is only phosphorylated by PKC in the modulation steps of the ACh release (Leenders and Sheng, 2005).

However, not always PKA and PKC cooperate in phosphorylating the same molecule or different subunits of the same complex. There are molecules and coupled functions modulated only by PKA. It seems that only PKA is involved in the desensitization induced by 5-HT in rat serotonergic neurons (Yao et al., 2010). Other molecules are modulated only by PKC. Spinal sigma-1 receptor-induced mechanical and thermal hypersensitivity are mediated by an increase in NO-induced PKC-dependent but PKA-independent expression of the spinal NMDA receptor GluN1 subunit (Roh et al., 2011). PKC isozymes modulate voltage-gated calcium (Ca_v_) currents through Ca_v_2.2 and Ca_v_2.3 channels by targeting serine/threonine (Ser/Thr) phosphorylation sites of Ca_v_α_1_ subunits. Stimulatory (Thr-422, Ser-2108 and Ser-2132) and inhibitory (Ser-425) sites were identified in the Ca_v_2.2α_1_ subunits to PKCs βII and ε. Net PKC effect may be the difference between the responses of the stimulatory and inhibitory sites (Rajagopal et al., 2017).

## Membrane Receptors and Serine Kinases

In most cells A_1_, M_1_ and TrkB operate mainly by stimulating the phospholipase C gamma (PLCγ) and, therefore, the PKC pathways and the inositol triphosphate (IP3) pathway, whereas A_2A_, M_2_ and M_4_ inhibit the adenyl cyclase (AC) and PKA pathway (Caulfield, 1993; Felder, 1995; Marala and Mustafa, 1995; Caulfield and Birdsall, 1998; Nathanson, 2000; De Lorenzo et al., 2004; Nishizaki, 2004; Oliveira and Correia-de-Sá, 2005).

**Figure 4:**
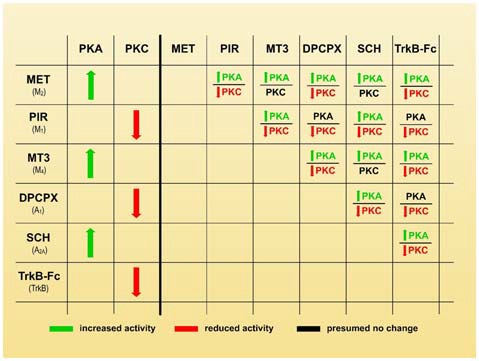
Membrane receptors and serine kinases. The left-hand side of the figure shows how the individual use of selective inhibitors changes protein kinase A (PKA) and protein kinase C (PKC) activity (green arrows mean stimulation, red arrows mean inhibition). The right-hand side of the figure shows that when two blockers are associated, PKA activity generally increases (green characters) or is unaffected in the cases of PIR-DPCPX, PIR-TrkB and DPCPX-TrkB associations (black characters). However, PKC activity is generally reduced (red characters) or unaffected in MET-MT3, MET-SCH58261 and MT3-SCH58261 dual inhibition (black characters also). Therefore, a higher PKA/PKC ratio can be produced by selective inhibitors and this coincide with a delay in axonal loss.

In considering the synergistic, antagonic and modulatory effects of the receptors on axonal loss (Figures 2, 3), we believe that an inhibition of PKA and/or stimulation of PKC in some nerve endings may play a leading role in promoting synapse elimination. Therefore, the left-hand side of Figure 4 shows how the individual use of selective inhibitors changes PKA and PKC activity in many cells (Caulfield, 1993; Felder, 1995; Calabresi et al., 1998; Caulfield and Birdsall, 1998; Nathanson, 2000; Santafé et al., 2006, 2007a; Salgado et al., 2007; Ansari et al., 2009; Tomàs et al., 2011; Rodrigues et al., 2014; Hughes et al., 2015; Obis et al., 2015). Theoretically, when two inhibitors are associated (right side of the Figure 4), PKA activity is generally increased (or unaffected in the cases of PIR-DPCPX, PIR-TrkB and DPCPX-TrkB associations, black characters). However, PKC activity is generally reduced (or unaffected in MET-MT3, MET-SCH58261 and MT3-SCH58261 dual inhibition, black characters). Therefore, the selective inhibitors would give a higher PKA/PKC ratio and delay axonal loss, which means that, in normal conditions without inhibitors, all the considered receptor pathways join together to give a lower PKA/PKC ratio and accelerate axonal loss.

However, although PKA and PKC are involved in synapse elimination, and changes in their respective activity seems relevant, a specific decrease of the PKA/PKC activity ratio would be not the best manner to describe their complementary role. Therefore, we hypothesize that “a membrane receptor-induced shifting in the PKA and PKC activity (inhibition of PKA and/or stimulation of PKC) in some nerve endings may play an important role in promoting developmental synapse elimination at the NMJ”.

In addition, the use of inhibitors show only the tonic effect of the molecule that is inhibited in basal conditions but the supposition that without the presence of the inhibitor the molecule play in all cases this tonic effect is a further deduction that will be considered as forming part of the hypothesis and analyzed with caution.

Although in 12 out of 15 simultaneous inhibitions with two drugs PKC activity is reduced and remains unchanged in only three (the same numbers applie for PKA activity increase and maintenance respectively, see Figure 4), it seems that a higher PKA/PKC ratio is the main factor in the paired receptors signaling inhibition. In this regard, there is no clear difference between the situations in which PKA presumably increases or is unchanged or when PKC decreases or remains unchanged in relation to axonal loss. This means that in paired inhibition conditions (two different receptors are blocked), the presumed relevant fact to influence axon loss seems to be the increase of the PKA activity only, the reduction of the PKC activity only or, in most cases, both situations simultaneously.

For instance, axon loss is also partially occluded between TrkB and both AR pathways (A_1_ and A_2A_) even when PKA would be not affected by blocking TrkB and A_1_ and PKA would increase by blocking TrkB and A_2A_. Also, a strong decrease in PKC while PKA remains stable can result in a synergistic effect of the inhibitors (PIR and DPCPX) or in an occlusion between them (DPCPX and TrkB). Therefore, the increase in the PKA/PKC ratio is the parameter that seems to change after all the direct and crossed inhibitions of the mAChR, AR and TrkB had been checked.

**Figure 5:**
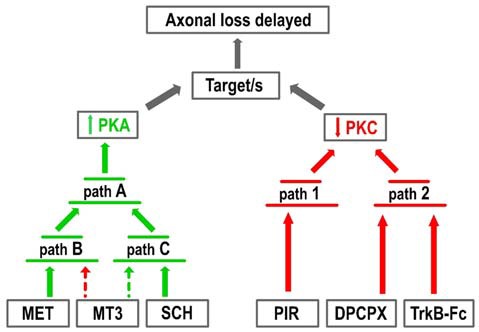
Pathways resulting in increased PKA and decreased PKC activity. The figure represents the two groups of receptor inhibitors: those that reduce PKC activity (right-hand side of the figure, red arrows) and those that increase PKA activity (left-hand side, green arrows). In terms of PKC, the effect of PIR is synergistic with the DPCPX and the TrkB-Fc effects although these last two substances are mutually occlusive. This suggests that PKC activity can be reduced by two parallel pathways (paths 1 and 2). In terms of PKA, the effects of MET and SCH58261 seem to converge on the final path A through the respective paths B and C, which cannot be summed even though they are in turn respectively modulated negatively (dotted red arrow) and positively (dotted green arrow) by MT3.

Figure 5 shows the two groups of receptor inhibitors separately: those that reduce PKC activity (right-hand side of the figure) and those that increase PKA activity (left-hand side). In terms of PKC, the effect of PIR is synergistic and can be added to the DPCPX and the TrkB-Fc effects although these last two substances are mutually occlusive. This suggests that PKC activity can be reduced by two parallel pathways (path 1 and 2 in the figure). Paths 1 and 2 can be summed but the pathways converging on path 2 cannot. In terms of PKA, the effects of MET and SCH58261 seem to converge on the final path A through their respective paths B and C, which cannot be summed even though they are in turn respectively modulated negatively and positively by MT3. Interestingly, a reduction in PKC (PIR) and an increase in PKA (SCH58261) can have a synergistic effect (Figure 2Ab). However, there is only one situation in which the inhibitors DPCPX (PKC reduction) and SCH58261 (PKA increase) fully antagonize each other (Figure 2Af). This suggests that, downstream of the AR, there is a common link that is inversely regulated by the two subtypes.

Therefore, in basal conditions, a reduction in PKA activity, an increase in PKC activity or, in most cases, both situations simultaneously, would accelerate synapse elimination.

There are many molecular targets of the membrane receptors-kinases phosphorylation pathways involved in transmitter release and nerve terminal stability. Their analysis is out of the scope here. However, during developmental axonal competition and loss, the nerve endings achieve differences in ACh release capacity and in the functional expression of several related molecules. Specifically, in the weakest endings (those that evoke small synaptic potentials) in polyinnervated NMJ, M_1_ receptors reduce release through the PKC pathway due to an excess of Ca^2+^ inflow through P-, N- and L-type calcium channels (L channel is only present in the weak endings). However, in the strongest and mature endings, the coupling of M_1_ to PKC activity results in ACh release potentiation using Ca^2+^ inflow through the P-channel. The PKA-linked M_2_ subtype is also present in the weakest endings, it is related only to P and N channels to potentiate release (Santafé et al., 2009a; see also Santafé et al., 2003, 2004, 2007a,b, 2009a,b; Tomàs et al., 2011). It is tempting to speculate on the relevance of the PKA and PKC phosphorylation of the Ca^2+^ channels in the differential control of transmitter release during axonal competition and nerve terminal loss.

## Conclusion and Hypothesis

We suggest that a membrane receptor-induced shifting in the PKA and PKC activity may play an important role in promoting developmental synapse elimination at the NMJ. This hypothesis is supported by: (i) the tonic effect (shown by using selective inhibitors) of several membrane receptors that accelerates axon loss between P5 and P9; (ii) the synergistic, antagonic and modulatory effects (shown by paired inhibition) of the receptors on axonal loss; (iii) the fact that the coupling of these receptors activates/inhibits the intracellular serine kinases; and (iv) the increase of the PKA activity, the reduction of the PKC activity or, in most cases, both situations simultaneously that presumably occurs in all the situations of singly and paired inhibition of the mAChR, AR and TrkB receptors.

The use of transgenic animals and various combinations of selective and specific PKA and PKC inhibitors could help to elucidate the role of these kinases in synapse maturation.

### Transgenic Mice

The transgenic mouse B6.Cg-Tg(Camk2a-Prkaca)426Tabe/J has a 50% reduction in basal cAMP-dependent PKA. Also, we found that nPKCε and cPKCβI isoforms are exclusively located in the motor nerve terminals of the adult rat NMJ and are involved in transmitter release (Besalduch et al., 2010; Lanuza et al., 2010; Obis et al., 2015). Thus, the use of the B6.129S4-*Prkce*^tm1Msg^/J mouse, homozygous for the *Prkce*^tm1Msg^ which is a nPKCε mutant mouse, may be useful.

### Selective and Specific PKA and PKC Modulators

The classic PKA antagonists H-89 and KT-5720 (De Lorenzo et al., 2006; Martinez-Pena y Valenzuela et al., 2013) and the agonist Dibutyryl-cAMP (Nelson et al., 2003) together with the PKC antagonists Calphostin C (CaC) and Go 6976 (Lanuza et al., 2002; Nili et al., 2006) and the PKC agonists phorbol 12-myristate 13-acetate and Bryostatin 1 (Lanuza et al., 2002; Sun and Alkon, 2006; Hage-Sleiman et al., 2015), will be useful tools. More importantly, the use of specific peptides that affect PKC translocation and activity may help us to understand what role these kinases play in axonal loss. For instance, the nPKCε-specific translocation inhibitor peptide, epsilon V1–2 (εV_1–2_; [Brandman et al., 2007; Obis et al., 2015]), the specific agonist peptide εV_1–7_ (Johnson et al., 1996), and the cPKCβI-specific translocation inhibitor peptide, betaI V5–3 (βIV_5–3_; Liu et al., 1999) together with the specific cPKCβI agonist dPPA (Rigor et al., 2010) will be helpful.

Exposure of these substances on the LAL surface during the synapse elimination period and counting the axons could be a simple and productive procedure (Nadal et al., 2016a,b, 2017).

## Ethics Approval

The mice were cared for in accordance with the guidelines of the European Community’s Council Directive of 24 November 1986 (86/609/EEC) for the humane treatment of laboratory animals. All experiments on animals have been reviewed and approved by the Animal Research Committee of the Universitat Rovira i Virgili (Reference number: 0233).

## Author Contributions

LN, EH, AS, VC and MT: data collection, quantitative analysis; literature search, data interpretation and graphic design; NG and MAL: statistics; JMT, NG and MAL: conception and design, literature search, data interpretation and manuscript preparation.

## Conflict of Interest Statement

The authors declare that the research was conducted in the absence of any commercial or financial relationships that could be construed as a potential conflict of interest.

## Abbreviations

AC: adenyl cyclase
ACh: acetylcholine
AR: adenosine receptors; A_1_adenosine receptor; A_2A_adenosine receptor
βIV_5–3_: translocation inhibitor peptide, beta I βIV_5–3_
CaC: calphostin C
Ca_v_: voltage-gated calcium
cPKCα: alpha protein kinase C isoform
cPKCβI: beta I protein kinase C isoform
DPCPX: 8-Cyclopentyl-1,3-IP3, inositol triphosphate
IP3: inositol triphosphate
LAL: Levator auris longus muscle
M_1_: M_1_-type muscarinic acetylcholine receptor
M_2_: M_2_-type muscarinic acetylcholine receptor
M_4_: M_4_-type muscarinic acetylcholine receptor
mAChR: muscarinic acetylcholine receptor
MET: methoctramine
nAChR: nicotinic acetylcholine receptor
nAChRδ: delta nicotinic acetylcholine receptor subunit
nAChRε: epsilon nicotinic acetylcholine receptor subunit
NMJ: neuromuscular junction
nPKCε: epsilon protein kinase C isoform
nPKCθ: theta protein kinase C isoform
NTR: neurotrophin receptor
OXO: oxotremorine
PIR: pirenzepine
PKA: protein kinase A
PKC: protein kinase C
PLC: phospholipase C
SCH58261: 2-(2-Furanyl)-7-(2-phenylethyl)-7*H*-pyrazolo[4,3-*e*][1,2,4]triazolo[1,5-*c*]pyrimidin-5-amine
TrkB: tropomyosin-related kinase B receptor
TrkB-Fc: inhibitor recombinant human TrkB-Fc Chimera.
